# Estrogen-modulating treatment among mid-life women and COVID-19 morbidity and mortality: a multiregister nationwide matched cohort study in Sweden

**DOI:** 10.1186/s12916-024-03297-z

**Published:** 2024-02-27

**Authors:** Evangelia Elenis, Helena Kopp Kallner, Maria A. Karalexi, David Hägg, Marie Linder, Katja Fall, Fotios C. Papadopoulos, Alkistis Skalkidou

**Affiliations:** 1 Department of Women’s and Children’s Health, Uppsala University, Uppsala, Sweden; 2 Reproduction Center, Women’s Clinic, Uppsala University Hospital, Uppsala, Sweden; 3 Department of Clinical Sciences at Danderyd Hospital, Karolinska Institutet, Stockholm, Sweden; 4 Department of Obstetrics and Gynecology, Danderyd Hospital, Stockholm, Sweden; 5 Department of Women’s and Children’s Health, Uppsala University, Uppsala, Sweden; 6 Department of Medicine Solna, Centre for Pharmacoepidemiology, Karolinska Institutet, Karolinska University Hospital, Stockholm, Sweden; 7 Clinical Epidemiology and Biostatistics, School of Medical Sciences, Faculty of Medicine and Health, Örebro University, Örebro, Sweden; 8 Unit of Integrative Epidemiology, Institute of Environmental Medicine, Karolinska Institutet, Stockholm, Sweden; 9 Department of Medical Sciences, Psychiatry, Uppsala University, Uppsala, Sweden

**Keywords:** Menopause hormonal treatments, Estrogens, Menopause, COVID-19, SARS-CoV-2

## Abstract

**Background:**

It has been repeatedly shown that men infected by SARS-CoV-2 face a twofold higher likelihood of dying, being hospitalized or admitted to the intensive care unit compared to women, despite taking into account relevant confounders. It has been hypothesized that these discrepancies are related to sex steroid hormone differences with estrogens being negatively correlated with disease severity. The objective of this study was therefore to evaluate COVID-19-related mortality and morbidity among peri- and postmenopausal women in relation to estrogen-containing menopause hormonal treatments (MHT).

**Methods:**

This is a national register-based matched cohort study performed in Sweden between January 1 to December 31, 2020. Study participants comprised women over the age of 53 years residing in Sweden. Exposure was defined as prescriptions of local estrogens, systemic estrogens with and without progestogens, progestogens alone, or tibolone. MHT users were then compared with a matched cohort of non-users. The primary outcome consisted of COVID-19 mortality, whereas the secondary outcomes included inpatient hospitalizations/outpatient visits and confirmed SARS-CoV-2 infection. Multivariable adjusted Cox regression-derived hazard ratios (HRs) were calculated.

**Results:**

Use of systemic estrogens alone is associated with increased COVID-19 mortality among older women (aHR 4.73, 1.22 to 18.32), but the association is no longer significant when discontinuation of estrogen use is accounted for. An increased risk for COVID-19 infection is further observed for women using combined systemic estrogens and progestogens (aHR 1.06, 1.00 to 1.13) or tibolone (aHR 1.21, 1.01 to 1.45). Use of local estrogens is associated with an increased risk for COVID-19-related death (aHR 2.02,1.45 to 2.81) as well as for all secondary outcomes.

**Conclusions:**

Systemic or local use of estrogens does not decrease COVID-19 morbidity and mortality to premenopausal background levels. Excess risk for COVID-19 morbidity and mortality was noted among older women and those discontinuing systemic estrogens. Higher risk for death was also noted among women using local estrogens, for which non-causal mechanisms such as confounding by comorbidity or frailty seem to be the most plausible underlying explanations.

**Trial registration details:**

Not applicable.

**Supplementary Information:**

The online version contains supplementary material available at 10.1186/s12916-024-03297-z.

## Background

Since the beginning of the coronavirus disease-19 (COVID-19) pandemic, several studies have aimed to comprehensively elucidate the biological pathways that underlie the vast differences in clinical course of the disease and which factors may influence these pathways. Globally, approximately 500 million people have been infected so far by the novel severe acute respiratory syndrome coronavirus 2 (SARS-CoV-2) with men being equally, if not slightly less, susceptible to infection than women [1–4]. However, men seem to experience almost two-fold higher mortality rates than women after controlling for potential confounding factors [5]. Similar patterns of disease severity have been observed with previous coronavirus outbreaks, including SARS-CoV (severe acute respiratory syndrome coronavirus) and MERS-CoV (Middle East respiratory syndrome coronavirus) [6–8].

The etiology of sex differences in COVID-19 severity and clinical course remains obscure. Several mechanisms have been hypothesized to underlie these differences including the role of sex hormones. One factor that has been suggested is the importance of estrogens in female immune responses, such as suppression of pro-inflammatory cytokines (i.e., IL-1β and IL-6), increased antibody production by B cells, and modulation of the activity of ACE2 [9, 10]. ACE2 is the receptor to which the virus’ spike protein binds when entering the cell. Although the exact mechanisms are yet to be understood, estrogens seem to play a role in preventing the cytokine storm release after a SARS-CoV-2 infection [9, 10]. In fact, accumulating data indicate that it is the cytokine storm and the subsequent respiratory distress and multiorgan failure that account for the high mortality observed among COVID-19 affected patients [11]. Biological sex differences in terms of sensitivity to severe COVID-19 may thus explain why fewer women than men have been hospitalized, have been admitted to intensive care units (ICU), and have died during the pandemic. However, only a handful of studies have yet examined the potential role of estrogen containing drugs on COVID-19 mortality [4, 12–15]. The existing studies indicate a protective effect of estrogen-containing medications against COVID-19 mortality among women already infected by SARS-CoV-2 [4, 12, 14]. However, all prior findings originate from data comprising combinations of exogenous menopausal hormone replacement therapy (MHT) with different drug preparations and administration routes, thus not allowing to delineate the potential effects of local versus systemic estrogen preparations, which often also refer to different dosages, as well as combinations with progestogens or not, against severe COVID-19. More evidence is needed to guide clinical recommendations.

The aim of this study is to evaluate COVID-19-related mortality and morbidity among peri- and postmenopausal women in relation to menopause hormonal treatments (MHT). This nationwide register-based study tests the hypothesis that MHT/estrogen-modulating treatment reduces COVID-19 mortality, disease severity, and risk for SARS-CoV-2 infection, among peri- and postmenopausal women in Sweden. Detailed register information allows for valid assessment of preparations containing estrogen, progestogen, and potential combinations, while also taking the hormone substance and administration route into account.

## Methods

### Study design

We performed a nationwide register-based matched cohort study in Sweden. Data were prospectively collected and retrieved pseudonymized after cross-linkages across different national socioeconomic and healthcare registries. Linkages were based on unique personal identification numbers of the study participants; all Swedish residents are assigned a unique 12-digit personal identification number at birth or upon immigration [16]. Data were assembled from the following registries: the National Patient Registry (NPR), including all in-patient care and outpatient specialists visits and related diagnoses from both private and public caregivers in Sweden [17]; the Prescribed Drug Registry (PDR), including information on the Anatomical Therapeutic Code (ATC-code), drug name, strength, date of prescription, and dispensing; the Cause of Death Registry (CDR), including information on cause of death, date, and place of death [18]; the Total Population Registry (TPR), including information on country of birth, sex, and marital status as well as immigration and emigration [19]; the Longitudinal Integration Database for Health Insurance and Labour Market Studies (LISA) [20] including information on educational attainment of the population; Sminet, a surveillance system collecting notifications for 60 communicable diseases classified either as *dangerous for public health* (such as HIV and hepatitis A–E) or *dangerous for society* (such as SARS-CoV-2 and Ebola) according to the Communicable Diseases Act [21]. Data from the National Patient Registry, the Prescribed Drug Registry, and the Cause of Death Registry were provided by the Swedish National Board of Health and Welfare, while the government agency Statistics Sweden provided data for the Total Population Registry and the Longitudinal Integration Database for Health Insurance and Labour Market Studies. The Public Health Agency of Sweden provided data for Sminet.

Patients and/or the public were not involved in this study.

### Study population

The study population comprises women residing in Sweden during January 1, 2020–December 31, 2020, who were 53 years or older at the study start, in order to increase the chance that only postmenopausal women were included in the population [22]. The exposed cohort comprises all women who had filled a prescription of any type of estrogen-modulating treatment with supply covering January 1, 2020, and potentially extending beyond this date. Information on estrogen-modulating treatment was retrieved from the PDR using ATC codes (Additional file 1: Appendix 1). For each exposed woman, up to five women without supply of any of these prescriptions of the exposed individual were randomly selected from the underlying population and matched by age (± 2 years) and healthcare region. Women from the comparison group could be matched to more than one exposed woman belonging to different exposure groups. Individuals with gender dysphoria diagnosed at any time during the study period and identified by the ICD-10-SE (International Classification of Diseases, tenth revision, Swedish Edition) codes F640, F648, and F649 were excluded.

**Fig. 1:**
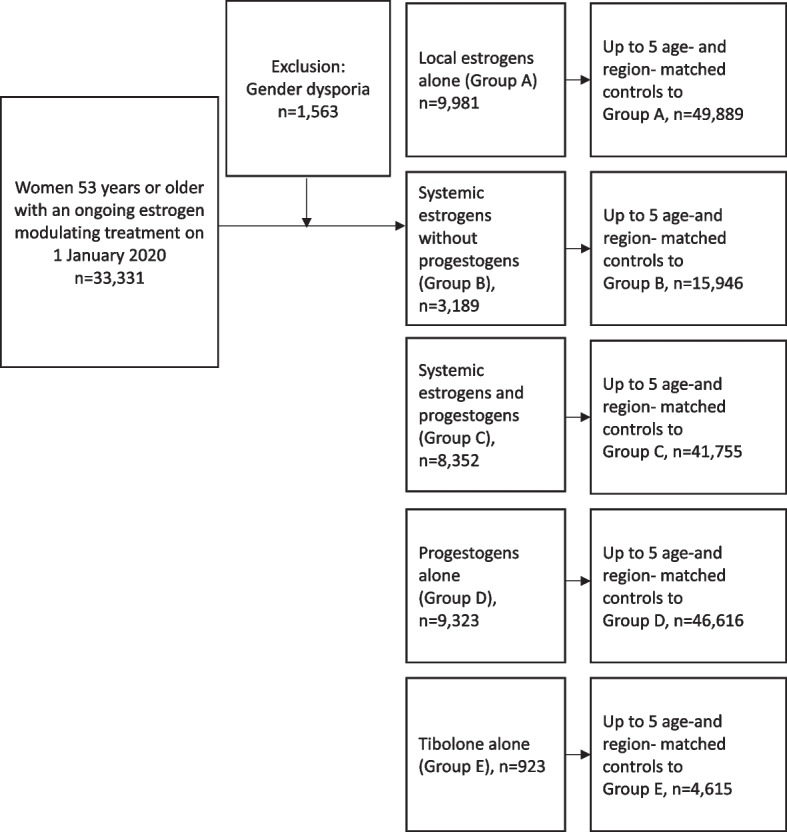
A flowchart displaying the included population

A flowchart displaying the included population is attached (Fig. 1).

### Study variables

#### Exposure

MHT use was defined as filled prescriptions of any hormonal modulating treatment for which the estimated duration overlapped with the study start (January 1, 2020). The duration of filled prescriptions was determined based on the defined daily dose (DDD) (i.e., the assumed average maintenance dose for a drug used for its main indication in adults) of vaginal, oral, transdermal, subcutaneous, or intramuscular tablets/gel/cream/spray/suppositories/patches/injections dispensed, allowing for a gap of 30 days between dispensing dates. Systemic estrogens comprised both oral as well as transdermal preparations, while progestogens included mostly synthetic progestins, but also rarely bioidentical progesterone and dydrogesterone. The use of hormonal intrauterine device (IUD) was defined as a prescription up to 5 years before first filled prescription date (5-year look-back). For filled prescriptions with missing data on defined daily dose (DDD), the duration was imputed from recommended use. The *ever exposure* approach was used, i.e., each woman was allocated to the exposure group starting at January 1, 2020, with that exposure continuing until censoring, regardless of following dispensing or discontinuation. If two or more of the specified drugs were prescribed during the same time-period, the drug with higher estrogenicity was used for categorization. Non-use was defined as no modulatory hormonal treatment supply at January 1, 2020 (with the exception of hormonal IUD).

The ATC codes utilized to define exposure state comprised in short: G03C (estrogens), G03F (continuous/sequential combination estrogen-progestogen), G03D (progestogens), G03AC (progestogens), G02BA (progestogens), or G03CX01 (tibolone) (for details, see Additional file 1: Appendix 1).

Exposure groups.

i) Women on local estrogens alone (group A)
ii) Women on systemic estrogens without progestogens (group B)
iii) Women on both estrogens and progestogens (group C)
iv) Women on progestogens alone (group D)
v) Women on tibolone alone (group E)

#### Follow-up and outcomes

The study participants were followed up for COVID-19 mortality or related morbidity until the first of either of the following events: death, emigration, study outcome, or the end of study (December 31, 2020).

The primary outcome was defined as death due to COVID-19 as either the main or underlying cause retrieved from the death certificates (according to the ICD-10-SE code U07.1 or U07.2). Secondary outcomes included the following: (i) inpatient hospitalizations (with or without the need for ICU admission) or outpatient visits due to COVID-19 (according to the ICD-10 codes U07.1 or U07.2) or (ii) laboratory COVID-19 confirmed events outside or inside a hospital setting (after having performed RT-PCR for SARS-CoV-2 from nasal or oropharyngeal swabs taken by healthcare personnel). Only outcomes registered after January 1, 2020, were considered valid. Data on the primary outcome of interest was retrieved from the CDR. Data on the secondary outcomes were retrieved from (i) the NPR and (ii) SmiNet, respectively. Outcomes were assessed independently; a person who was admitted in the hospital and then died from COVID-19 was included both in the death event counts (primary outcome) and in the inpatient hospitalizations/outpatient visits event counts (secondary outcome). Similar imputations were made within the secondary outcomes, i.e., all registered inpatient hospitalizations/outpatient visits events were included among the laboratory COVID-19 confirmed events.

#### Covariates

Data on the following sociodemographic factors were retrieved from the TPR and LISA registers: age at study inclusion (years), civil status (married/register partnership *vs* unmarried), income (low [below 20th percentile], middle [21st–79th percentile], or high [80th percentile and above] in relation to the income distribution in Sweden) and education level (< 9, 9–12, 13 years or more of total education attained). Regarding potential comorbidities and their effect on the outcome of interest a composite proxy, the modified Charlson Comorbidity Index was utilized. The Charlson Comorbidity Index (CCI) is a disease index often used as a proxy for comorbidity burden in statistical analysis. It is used to categorize comorbidities based on the ICD-10 codes of certain comorbid conditions recorded in the NPR (data included between January 1, 2016, through January 1, 2020). Each comorbidity category has an associated weight based on the adjusted risk of 1-year mortality, the sum of which results in a single comorbidity score for each patient. For the calculation of the modified CCI in our study, we included data based on COVID-19-related risk factors only. A complete list with the diagnoses and weights employed in the analysis is included in Additional file 1: Appendix 2. Proxies were used for alcohol abuse (ICD-10 code F10.2) and obesity (ICD-10 code E66) and were accounted for as independent risk factors for COVID-19 complications [23].

### Statistical analysis

#### Main analysis

We first calculated unadjusted incidence rates (number of outcomes divided by accumulated person-years) of COVID-19-related mortality and morbidity among women with and without hormonal treatment. Rates of outcomes and covariates were calculated for different exposure groups. Cox regression analyses were performed in order to estimate hazard ratios with 95% confidence intervals (CIs) for the primary and secondary outcomes in relation to the exposure status. Regression models were adjusted for age at study inclusion, civil status, income, education level, obesity, alcohol abuse, and modified CCI. We have also repeated the regression analysis after adjusting for CCI categories as independent factors instead of as a composite proxy variable. Sensitivity analyses were performed in order to explore the impact of age as well as ongoing treatment on COVID-19 primary and secondary outcomes. Firstly, the analyses were repeated after stratifying the different exposure groups by age group at inclusion (i.e., 53–62 years, 63–72 years, and 73 years or over). Lastly, we studied the effect on all-cause mortality of “current ongoing treatment only” by censoring exposed women when they discontinued treatment or reached the end of supply before the end of the study (i.e., restricting the analyses among those with ongoing treatment as opposed to ever treatment). A two-sided *p*-value below 0.05 was considered statistically significant. All statistical analyses were performed using Statistical Analysis Software (SAS) version 9.4 (SAS Institute, Cary, NC, USA).

### Further analyses

In order to evaluate the impact of different factors affecting the COVID-19 disease severity, we conducted the following explorative analyses:

- The impact on the primary and secondary outcomes of all preparations containing systemic estrogen with and without progestogen grouped together
- The competing risks effect, by assessing also all-cause mortality
- The effect of timing of COVID-19 morbidity and mortality in relation to the COVID-19 pandemic outbreak, by stratifying the primary and secondary outcomes in relation to the first (1 January 2020–31 August 2020) or second wave of COVID-19 pandemic (1 September 2020–31 December 2020)
- The impact of the route of administration of systemic estrogens, i.e., oral *vs* transdermal estrogen-including MHT, on the primary outcome

## Results

### Demographic characteristics

The exposed groups included 9981 women using local estrogens alone (group A), 3189 on systemic estrogens without progestogens (group B), 8352 on estrogens and progestogens (group C), 9323 on progestogens alone (group D), and 923 women receiving tibolone alone (group E) (Table 1). Mean age of the study population was 71.4 years (± standard deviation [SD]: 11.2) for group A, 58.2 (± 6.5) for group B, 57.2 (± 4.8) for group C, 55.8 (± 4.9) for group D, and 59.8 (± 6.0) for group E and was similar between the different exposure groups and corresponding comparison groups. A higher proportion (around 50%) of women exposed to systemic estrogen and/or progestogen (groups B-D) had attained more than 13 years of education compared with women in the matched cohort (42%), while no differences were observed between the comparison cohort and women using local estrogens alone or tibolone alone. Fewer women on systemic estrogen treatment or tibolone alone (groups B–C, E) had received a diagnosis of obesity compared with the matched cohort. Lastly, the majority (around 88%) of both exposed and unexposed women were healthy at baseline as indicated by a zero Charlson Comorbidity Index (CCI) with no numerical differences noted between the groups (Table 1).

**Table 1:** Demographic characteristics of the study population, exposed women, and matched unexposed women presented by drug preparation and route of administration

	**Local estrogens alone**		**Systemic estrogens without progestogens**		**Estrogens and progestogens**		**Progestogens alone**		**Tibolone alone**	
	**Exposed**	**Unexposed**	**Exposed**	**Unexposed**	**Exposed**	**Unexposed**	**Exposed**	**Unexposed**	**Exposed**	**Unexposed**
**Person time (in years)**	9438	47,183	3100	15,505	8341	41,693	9272	46,356	922	4612
**Age (years),** mean (SD)	71.4 (11.2)	71.4 (11.2)	58.2 (6.5)	58.2 (6.5)	57.2 (4.8)	57.2 (4.8)	55.8 (4.9)	55.8 (4.9)	59.8 (6.0)	59.8 (6.0)
Median, [Q1–Q3]	71.0 (62.0–79.0)	71.0 (62.0–79.0)	56.0 (54.0–59.0)	56.0 (54.0–59.0)	56.0 (54.0–58.0)	56.0 (54.0–58.0)	54.0 (53.0–56.0)	54.0 (53.0–56.0)	58.0 (56.0–62.0)	58.0 (56.0–62.0)
**Age group (years)** *n* (%)										
53–62	2577 (25.8)	12885 (25.8)	2715 (85.1)	13,576 (85.1)	7508 (89.9)	37,535 (89.9)	8803 (94.4)	44,016 (94.4)	699 (75.7)	3495 (75.7)
63–72	2976 (29.8)	14,880 (29.8)	308 (9.7)	1540 (9.7)	654 (7.8)	3270 (7.8)	316 (3.4)	1580 (3.4)	175 (19.0)	875 (19.0)
> 73	4428 (44.4)	22,124 (44.3)	166 (5.2)	830 (5.2)	190 (2.3)	950 (2.3)	204 (2.2)	1020 (2.2)	49 (5.3)	245 (5.3)
**Education (years),** *n* (%)										
< 9	1321 (13.2)	6624 (13.3)	55 (1.7)	361 (2.3)	46 (0.6)	707 (1.7)	75 (0.8)	785 (1.7)	9 (1.0)	97 (2.1)
9	824 (8.3)	3836 (7.7)	158 (5.0)	984 (6.2)	439 (5.3)	2681 (6.4)	391 (4.2)	2853 (6.1)	59 (6.4)	313 (6.8)
10–12	4228 (42.4)	21,659 (43.4)	1356 (42.5)	7739 (48.5)	3931 (47.1)	20,584 (49.3)	4448 (47.7)	23,230 (49.8)	434 (47.0)	2176 (47.29
12 +	3525 (35.3)	17,147 (34.3)	1607 (50.4)	6788 (42.6)	3925 (47.0)	17,637 (42.2)	4379 (47.0)	19,566 (42.0)	421 (45.6)	2001 (43.4)
Unknown	83 (0.8)	623 (1.2)	13 (0.4)	74 (0.5)	11 (0.1)	146 (0.3)	30 (0.3)	182 (0.4)	0 (0.0)	28 (0.6)
**Civil status,** *n* (%)										
Married/registered partner	4481 (44.9)	22,350 (44.8)	1429 (44.8)	7144 (44.8)	3742 (44.8)	18,748 (44.9)	4177 (44.8)	20,931 (44.9)	413 (44.7)	2072 (44.9)
**Income,** *n* (%)										
Low	4931 (49.4)	24,396 (48.9)	3189 (49.2)	15,946 (49.7)	4109 (49.2)	20,669 (49.5)	9323 (49.3)	46,616 (49.4)	923 (49.3)	2280 (49.4)
Middle	2337 (47.4)	11,564 (47.4)	1569 (47.3)	7925 (46.9)	1935 (47.1)	9756 (47.2)	4596 (47.1)	23,028 (47.0)	455 (46.9)	1074 (47.1)
High	75 (3.2)	428 (3.7)	742 (3.5)	3717 (3.4)	72 (3.7)	322 (3.3)	2165 (3.6)	10,823 (3.6)	213 (3.8)	38 (3.5)
**Obesity,** *n* (%)	155 (1.6)	721 (1.4)	41 (1.3)	342 (2.1)	96 (1.1)	839 (2.0)	246 (2.6)	1011 (2.2)	5 (0.5)	80 (1.7)
**Alcohol dependence,** *n* (%)	46 (0.5)	116 (0.2)	17 (0.5)	56 (0.4)	25 (0.3)	162 (0.4)	47 (0.5)	209 (0.4)	5 (0.5)	22 (0.5)
**Charlson Comorbidity Index** *n* (%)										
0	8773 (87.9)	43,952 (88.1)	2813 (88.2)	14,001 (87.8)	7358 (88.1)	37,037 (88.7)	8270 (88.7)	41,115 (88.2)	810 (87.8)	4061 (88.0)
1	1198 (12.0)	5887 (11.8)	348 (10.9)	1770 (11.1)	986 (11.8)	4677 (11.2)	1044 (11.2)	5454 (11.7)	113 (12.2)	554 (11.9)
2 +	10 (0.1)	50 (0.1)	29 (0.9)	175 (1.1)	8 (0.1)	42 (0.1)	9 (0.1)	47 (0.1)	0 (0.0)	0 (0.0)

### COVID-19 mortality

During follow-up, 114 cases of COVID-19 deaths were observed among the unexposed women and 50 among women who had used local estrogens (incidence rate, 5.3 per 1000 person-years) (Table 2), yielding a statistically significant hazard ratio of 2.02 (95% CIs: 1.45–2.81) for COVID-19 mortality in the adjusted models (Table 3). Women treated with systemic estrogens without progestogens also showed a statistically significant increased risk of COVID-19-related death compared with matched unexposed individuals (adjusted HR: 6.39, 95% CIs: 1.69–24.21). No deaths due to COVID-19 were reported for women treated with a combination of estrogens and progestogens or with tibolone alone with similar death rates estimated in the matched unexposed population. No clear difference in mortality risk was observed for women using progestogens alone compared with non-users (Table 3).

**Table 2:** Incidence rates (IR) for the association of sex steroid treatment with all primary and secondary study outcomes

	**Laboratory confirmed SARS-CoV-2 infection**			**Outpatient visits/inpatient hospitalizations with/without ICU admission**		**Death due to COVID-19**	
**Exposure**	**Number of persons**	**Median follow-up (years)**	**IR per 1000 person-years (95% CIs)**	**Median follow-up (years)**	**IR per 1000 person-years (95% CIs)**	**Median follow-up (years)**	**IR per 1000 person-years (95% CIs)**
Local estrogens alone	9981	0.99863	111.6 (104.9–118.6)	0.96	41.3 (37.2–45.6)	0.999	5.3 (3.9–7.0)
Systemic estrogens without progestogens	3189	0.99863	138.2 (125.4–152.1)	0.97	23.5 (18.4–29.6)	0.999	1.6 (0.5–3.7)
Estrogens and progestogens	8352	0.99863	145.2 (137.1–153.7)	0.96	14.8 (12.3–17.7)	0.999	0.0 (0.0–0.0)
Progestogens alone	9323	0.99863	150.1 (142.3–158.2)	0.96	18.3 (15.6–21.2)	0.999	0.2 (0.0–0.8)
Tibolone alone	923	0.99863	156.0 (131.6–156.0)	0.98	25.2 (16.0–37.8)	0.999	0.0 (0.0–0.3)

*Abbreviations*: *IR* Incidence rates, *CI* Confidence intervals

**Table 3:** Crude and adjusted hazard ratios (HRs) for the association of sex steroid treatment with death due to COVID-19

	**Exposed**		**Unexposed**			
**Exposure**	**Total person-time (in years)**	***n* events (%)**	**Total person-time (in years)**	***n* events (%)**	**Crude HR (95% CI)**	**Adjusted HR (95% CI)**^**a**^
Local estrogens alone	9438	50 (0.50)	47,146	114 (0.23)	2.19 (1.57–3.06)	2.02 (1.45–2.81)
Systemic estrogens without progestogens	3100	5 (0.16)	15,503	4 (0.03)	6.26 (1.68–23.30)	6.39 (1.69–24.21)
Estrogens and progestogens	8341	0 (0.00)	41,692	4 (0.01)	-	-
Progestogens alone	9272	2 (0.02)	46,354	7 (0.02)	1.43 (0.30–6.88)	1.95 (0.41–9.42)
Tibolone alone	922	0 (0.00)	4615	0 (0.00)	-	-

*Abbreviations*: *HR* Hazard ratio, *CI* Confidence intervals

^**a**^Models adjusted for age, civil status, income, education, obesity, alcohol dependence, and Charlson Comorbidity Index

### COVID-19 morbidity and SARS-CoV-2 infection

Women using local estrogens alone experienced higher risk of outpatient visits/inpatient hospitalizations with or without the need for ICU admission (aHR: 1.23, 95% CIs: 1.10–1.38) and higher risk of SARS-CoV-2 infection (aHR: 1.13, 95% CIs: 1.06–1.21; Table 4). In the remaining exposed groups, no associations were found for the secondary outcomes of interest. The only exception was a significantly higher risk of COVID-19 infection for women exposed both to estrogens and progestogens (aHR: 1.06, 95% CIs: 1.00–1.13) and for women exposed to tibolone alone (aHR: 1.23, 95% CIs: 1.01–1.45; Table 4) compared with their matched non-exposed women.

**Table 4:** Crude and adjusted Cox regression models regarding the association of sex steroid treatment with laboratory confirmed SARS-CoV-2 infection or outpatient visits/inpatient hospitalizations with or without the need for ICU admission due to COVID-19

	**Laboratory confirmed SARS-CoV-2**						**Outpatient visits/inpatient hospitalizations with/without ICU admission**					
	**Exposed**		**Unexposed**				**Exposed**		**Unexposed**			
**Exposure**	**Person-time (years)**	***n* events (%)**	**Person-time (years)**	***n* events (%)**	**Crude HR (95% CI)**	**Adjusted HR (95% CI)**	**Person-time (years)**	***n* events (%)**	**Person-time (years)**	***n* events (%)**	**Crude HR (95% CI)**	**Adjusted HR (95% CI)**
Local estrogens alone	9346	1043 (10.45)	46,775	4552 (9.12)	1.15 (1.07–1.23)	1.13 (1.06–1.21)	9308	384 (3.85)	46,614	1523 (3.05)	1.26 (1.13–1.41)	1.23 (1.10–1.38)
Systemic estrogens without progestogens	3066	424 (13.30)	15,299	2211 (13.87)	0.96 (0.87–1.07)	0.97 (0.88–1.08)	3067	72 (2.26)	15,359	350 (2.19)	1.03 (0.80–1.33)	1.05 (0.82–1.36)
Estrogens and progestogens	8237	1196 (14.32)	41,153	5615 (13.45)	1.06 (1.00–1.13)	1.06 (1.00–1.13)	8289	123 (1.47)	41,360	776 (1.86)	0.79 (0.66–0.96)	0.84 (0.69–1.01)
Progestogens alone	9148	1373 (14.73)	45,765	6601 (14.16)	1.04 (0.98–1.10)	1.03 (0.97–1.09)	9199	168 (1.80)	45,972	913 (1.96)	0.92 (0.78–1.08)	0.95 (0.81–1.13)
Tibolone alone	923	144 (15.60)	4615	589 (12.76)	1.23 (1.02–1.47)	1.21 (1.01–1.45)	912	23 (2.49)	4573	94 (2.04)	1.23 (0.78–1.93)	1.27 (0.80–2.00)

*Abbreviations*: *HR* Hazard ratio, *CI* Confidence intervals

Models adjusted for age, civil status, income, education, obesity, alcohol dependence, and Charlson Comorbidity Index

### Sensitivity analyses

Analyses of different age groups showed that the risk of death due to COVID-19 was significantly increased for women above 63 years of age receiving local estrogens alone, with women aged 63–72 years exhibiting the highest risk (aHR 4.17, 95% CIs: 1.70–13.65). Only older women (i.e., 73 years or above) using systemic estrogens without progestogens exhibited increased risk of dying due to COVID-19 (aHR 4.73, 95% CIs: 1.22–18.32) (Tables 5 and 6). When only considering ongoing treatment, the positive association with COVID-19 mortality was no longer significant for systemic estrogens (Tables 7 and 8) and remained significant only for local estrogens alone (aHR 1.71, 95% CIs: 1.03–2.82).

**Table 5:** Crude and adjusted Cox regression models stratified by age regarding the association of sex steroid treatment with death due to COVID-19

Death due to COVID-19							
		**Exposed**		**Unexposed**			
**Exposure**	**Age (years)**	***n* total**	***n* events (%)**	***n* total**	***n* events (%)**	**Crude HR (95% CI)**	**Adjusted HR (95% CI)**^a^
Local estrogens alone	53–62	2577	0 (0.00)	12,885	0 (0.00)	NE	NE
	63–72	2976	5 (0.17)	14,880	6 (0.04)	4.17 (1.27–13.65)	4.33 (1.31–14.30)
	> 73	4428	45 (1.02)	22,124	108 (0.49)	2.08 (1.47–2.95)	1.87 (1.32–2.65)
Systemic estrogens without progestogens	53–62	2715	0 (0.00)	13,576	0 (0.00)	NE	NE
	63–72	308	0 (0.00)	1540	0 (0.00)	NE	NE
	> 73	166	5 (3.01)	830	4 (0.48)	6.25 (1.68–23.27)	4.73 (1.22–18.32)
Systemic estrogens and progestogens	53–62	7508	0 (0.00)	37,535	1 (0.00)	NE	NE
	63–72	654	0 (0.00)	3270	1 (0.03)	NE	NE
	> 73	190	0 (0.00)	950	2 (0.21)	NE	NE
Progestogens alone	53–62	8803	0 (0.00)	44,016	1 (0.00)	NE	NE
	63–72	316	0 (0.00)	1580	0 (0.00)	NE	NE
	> 73	204	2 (0.98)	1020	6 (0.59)	1.67 (0.34–8.26)	2.03 (0.40–10.43)
Tibolone alone	53–62	699	0 (0.00)	3495	0 (0.00)	NE	NE
	63–72	175	0 (0.00)	875	0 (0.00)	NE	NE
	> 73	49	0 (0.00)	245	0 (0.00)	NE	NE

*Abbreviations*: *HR* Hazard ratio, *CI* Confidence intervals, *ICU* Intensive care unit, *NE* Not estimated

^a^Models adjusted for occupation, family type, education, obesity, alcohol dependence syndrome, and Charlson Comorbidity Index

**Table 6:** Crude and adjusted Cox regression models stratified by age regarding the association of sex steroid treatment with laboratory confirmed SARS-CoV-2 infection or outpatient visits/ inpatient hospitalizations with or without the need for ICU admission due to COVID-19

		**Laboratory confirmed SARS-CoV-2**						**Outpatient visits/Inpatient hospitalizations with/without ICU admission**					
		**Exposed**		**Unexposed**				**Exposed**		**Unexposed**			
**Exposure**	**Age**	***n* total**	***n* events (%)**	***n* total**	***n* events (%)**	**Crude HR (95% CI)**	**Adjusted HR (95% CI)**	***n* total**	***n* events (%)**	***n* total**	***n* events (%)**	**Crude HR (95% CI)**	**Adjusted HR (95% CI)**
Local estrogens alone	53–62	2577	443 (4.43)	12,885	2077 (4.16)	1.05 (0.93–1.18)	1.07 (0.95–1.20)	2577	68 (0.68)	12,885	353 (0.71)	1.13 (0.67–1.92)	1.13 (0.67–1.92)
	63–72	2976	271 (2.71)	14,880	1158 (2.32)	1.18 (1.01–1.37)	1.17 (1.00–1.36)	2976	97 (0.97)	14,880	311 (0.62)	2.21 (1.41–3.48)	2.21 (1.41–3.48)
	> 73	4428	330 (3.30)	22,124	1316 (2.64)	1.25 (1.09–1.43)	1.19 (1.04–1.37)	4428	219 (2.19)	22,124	858 (1.72)	1.60 (1.18–2.19)	1.60 (1.18–2.19)
Systemic estrogens without progestogens	53–62	2715	392 (12.3)	13,576	2076 (13.02)	0.93 (0.83–1.04)	0.94 (0.84–1.06)	2715	64 (2.00)	13,576	300 (1.88)	0.92 (0.55–1.54)	0.92 (0.55–1.54)
	63–72	308	20 (0.64)	1540	100 (0.62)	0.99 (0.60–1.64)	1.01 (0.61–1.69)	308	3 (0.09)	1540	30 (0.19)	NE	NE
	> 73	166	11 (0.35)	830	35 (0.22)	1.47 (0.73–2.98)	1.38 (0.67–2.82)	166	6 (0.17)	830	19 (0.12)	0.83 (0.10–6.92)	0.83 (0.10–6.92)
Systemic estrogens and progestogens	53–62	7508	1141 (13.66)	37,535	5360 (12.84)	1.08 (1.01–1.15)	1.07 (1.00–1.15)	7508	111 (1.33)	37,535	691 (1.65)	0.72 (0.51–1.03)	0.72 (0.51–1.03)
	63–72	654	47 (0.57)	3270	223 (0.54)	1.08 (0.78–1.49)	1.11 (0.80–1.54)	654	12 (0.14)	3270	57 (0.14)	1.14 (0.43–3.00)	1.14 (0.43–3.00)
	> 73	190	8 (0.09)	950	31 (0.08)	1.17 (0.51–2.66)	1.18 (0.51–2.71)	190	0 (0.00)	950	28 (0.07)	NE	NE
Progestogens alone	53–62	8803	1344 (14.41)	44,016	6440 (13.81)	1.04 (0.98–1.11)	1.03 (0.97–1.10)	8803	154 (1.65)	44,016	867 (1.86)	0.74 (0.54–1.01)	0.74 (0.54–1.01)
	63–72	316	15 (0.16)	1580	108 (0.23)	0.70 (0.40–1.23)	0.68 (0.38–1.23)	316	0 (0.00)	1580	13 (0.03)	NE	NE
	> 73	204	14 (0.15)	1020	53 (0.11)	1.43 (0.79–2.59)	1.48 (0.77–2.84)	204	14 (0.16)	1020	32 (0.07)	2.50 (0.75–8.30)	2.50 (0.75–8.30)
Tibolone alone	53–62	699	119 (12.90)	3495	509 (11.03)	1.20 (0.97–1.47)	1.19 (0.97–1.47)	699	17 (1.87)	3495	80 (1.73)	0.77 (0.27–2.21)	0.77 (0.27–2.21)
	63–72	175	24 (2.58)	875	72 (1.56)	1.80 (1.12–2.89)	1.82 (1.12–2.95)	175	3 (0.31)	875	9 (0.19)	1.25 (0.14–11.18)	1.25 (0.14–11.18)
	> 73	49	1 (0.12)	245	8 (0.17)	0.63 (0.08–5.00)	0.70 (0.09–5.72)	49	3 (0.31)	245	6 (0.12)	2.50 (0.23–27.57)	2.50 (0.23–27.57)

*Abbreviations*: *HR* Hazard ratio, *CI* Confidence intervals, *ICU* Intensive care unit, *NE* Not estimated

Models adjusted for occupation, family type, education, obesity, alcohol dependence syndrome, and Charlson Comorbidity Index

**Table 7:** Crude and adjusted Cox regression models evaluating the effect of treatment cessation in relation to death due to COVID-19 (i.e., restricting the analyses among those with ongoing treatment as opposed to ever treatment)

Death due to COVID-19						
	**Exposed**		**Unexposed**			
**Exposure**	**Person time (in years)**	***n* events (%)**	**Person time (in years)**	***n* events (%)**	**Crude HR (95% CI)**	**Adjusted HR (95% CI)**
Local estrogens alone	3964	21	19,813	55	1.55 (0.94–2.56)	1.71 (1.03–2.82)
Systemic estrogens without progestogens	1240	2	6200	0	NE	NE
Systemic estrogens and progestogens	3248	0	16,236	0	NE	NE
Progestogens alone	4636	1	23,180	2	0.96 (0.09–10.57)	0.65 (0.05–7.83)
Tibolone alone	359	0	1794	0	NE	NE

**Table 8:** Crude and adjusted Cox regression models evaluating the effect of treatment cessation in relation to laboratory confirmed SARS-CoV-2 infection or outpatient visits/inpatient hospitalizations with or without the need for ICU admission (i.e., restricting the analyses among those with ongoing treatment as opposed to ever treatment)

	**Laboratory confirmed SARS-CoV-2**						**Outpatient visits/inpatient hospitalizations with/without ICU admission**					
	**Exposed**		**Unexposed**				**Exposed**		**Unexposed**			
**Exposure**	**Person time (in years)**	***n* events (%)**	**Person time (in years)**	***n* events (%)**	**Crude HR (95% CI)**	**Adjusted HR (95% CI)**	**Person time (in years)**	***n* events (%)**	**Person time (in years)**	***n* events (%)**	**Crude HR (95% CI)**	**Adjusted HR (95% CI)**
Local estrogens alone	3962	192	19,814	513	1.51 (1.28–1.78)	1.50 (1.26–1.75	3962	135	19,812	510	1.07 (0.89–1.30)	1.12 (0.93–1.36)
Systemic estrogens without progestogens	1238	128	6201	130	0.91 (0.71–1.16)	0.93 (0.73–1.19)	1242	45	6201	21	1.97 (1.17–3.30)	1.99 (1.18–3.35)
Estrogens and progestogens	3247	365	16,252	794	1.08 (0.95–1.22)	1.07 (0.94–1.21)	3249	73	16,233	188	089 (0.68–1.17)	0.91 (0.69–1.19)
Progestogens alone	4629	1035	23,178	1996	0.96 (0.89–1.04)	0.97 (0.90–1.04)	4635	148	23,178	279	0.99 (0.81–1.21)	1.00 (0.82–1.22)
Tibolone alone	358	45	1793	14	0.90 (0.49–1.63)	0.83 (0.45–1.53)	355	14	1791	3	1.31 (0.38–4.58)	1.44 (0.41–5.09)

### Further analyses

- Regardless of concomitant progestogen use or route of administration, we found that systemic estrogens conferred an increased risk of death by COVID-19 (aHR 4.22, 95% CIs: 1.37–13.05), but there was no apparent effect on the secondary outcomes (*p* > 0.05) (Additional file 2: Table S1).
- When assessing all-cause mortality, no differences were observed between exposure groups and matched comparison groups (Additional file 2: Table S2).
- With regard to the timing of the outbreak of the pandemic, we observed that the associations with COVID-19 morbidity and mortality were statistically significant mostly during the first wave and not consistently significant during the second wave (Additional file 2: Table S3).
- With regard to the route of administration of systemic estrogens without progestogens, we observed an increased risk of death from COVID-19 only among users of oral estrogens with an incidence rate of 4.6 (1.05–10.7), while no estimation could be performed among the users of transdermal preparations as there were no deaths observed in that group during the study period.

Throughout the analyses, in models using the distinct CCI contributors instead of the composite index, results remained substantially unchanged (data not shown).

## Discussion

Overall, the present large Swedish cohort of peri- and postmenopausal women does not demonstrate any reduced risk for COVID-19 infection or related mortality among women using MHT compared with unexposed women, suggesting that MHT was not able to reverse theoretical increased risks among women with vasomotor symptoms or genitourinary symptoms*.* Initiating or continuing MHT treatment solely as a prophylactic treatment against COVID-19 should therefore be avoided. On the contrary, women on local estrogen alone experienced higher COVID-19 mortality, higher risk of outpatient visits/inpatient hospitalizations, and higher risk of SARS-CoV-2 infection. Also, there was an increased risk for death in the group on systemic estrogens without progestogens, but the excess risk was observed primarily in elderly women, and those discontinuing estrogen therapy during the follow-up period. When assessing mortality rates among women on ongoing estrogen therapy, no increased risk could be seen. Indication bias and residual confounding by co-morbidities not available to control for could thus at least partially explain these associations. In addition, the associations between estrogen use and COVID-19 outcomes were only observed during the first wave of the pandemic, further suggesting that non-causal mechanisms may explain the observed excess risks.

### Interpretation

Increased risk of death from COVID-19 in women treated with systemic estrogens with or without progestogens as well as local estrogens have not been reported before. These findings, at first look, seem to disagree with what would be expected based on pathophysiological mechanisms and findings from the limited existing literature and were admittedly unanticipated. However, in a sensitivity analysis, we observed that the association between systemic estrogen therapy and COVID-19 mortality was no longer significant when exposure was defined as ongoing treatment with systemic estrogen therapy and not ever-treatment. The unfavorable effect of systemic estrogens on COVID-19 mortality may thus be explained by estrogen withdrawal rather than estrogen continuation, an observation that is in line with what other studies have so far demonstrated. Indeed, this fraction of peri- and postmenopausal women receiving MHT treatment probably represent a population of lower baseline estrogen levels who however restore their levels to those found in pre-menopause during treatment, i.e., a period in life with lower risk for severe COVID-19. However, when these women interrupted their treatment, their hormone levels fell to postmenopausal levels, and it is only then that the undesired severe effects of COVID-19 were noted. The latter observation strengthens the notion for a protective role of estrogens against COVID-19 and is in agreement with the literature, possibly implying that the relationship between COVID-19 and sex hormones is more complex than initially hypothesized. Although the exact mechanism is still unclear, it is believed that estrogen receptor a (ERα) directly interacts with the spike protein of the SARS-CoV-2 in certain tissues leading to a modified signaling pathway, which potentially affects SARS-CoV-2 infection and related pathology [24].

Estrogens were highlighted already from the beginning of the pandemic as playing a central role in COVID-19 morbidity and mortality due to the increased risk of deep vein thrombosis in COVID-19 infected individuals [25]. Thus, some exposed women at higher risk might have taken the initiative to discontinue their medications, especially systemic estrogen-containing preparations, during the study period, inducing a risk of misclassification. Likewise, women with systemic estrogen containing treatments might have been urged by their prescribing physician to seek medical attention earlier or to a greater extent if having COVID-19 like symptoms inducing a risk of over-ascertainment of some secondary outcomes (i.e., laboratory confirmed SARS-CoV-2 infection), explaining the higher risk for infection noted in some groups (surveillance bias). It is therefore plausible and important to consider that some of the associations noted between estrogen-modulating treatment and COVID-19 disease could also partly be explained by different types of bias which could not be controlled for in this study design, rather than suggesting causality and not by the treatment itself. It is of note that treatment with systemic estrogens and progestogens as well as tibolone exhibited neither a protective nor a harmful effect.

Furthermore, a higher risk for all morbidity outcomes as well as COVID-19 mortality were noted for women treated solely with local estrogens. It is generally known that the estrogen plasma levels that are reached due to local estrogen use are usually very low and do not differ greatly from the levels reported in healthy untreated postmenopausal women (i.e., 4 pg/ml increase in estradiol levels during use) [26]; one would therefore not expect a pharmacologic effect systemically. Local estrogens are usually prescribed against genitourinary symptoms of menopause. In fact, menopausal symptoms, both vasomotor and genitourinary, have been reported to reflect an underlying estrogen deficiency and endothelial dysfunction [27, 28], making these women more susceptible for cardiovascular events and complications [29, 30]. Moreover, women with menopausal symptoms, especially vaginal atrophy, are more prone to local infections (e.g., urinary tract infection (UTI), recurrent candidiasis etc.) [31, 32] with newer data indicating a link even to systemic infections, among which COVID-19 [15, 33, 34]; we could therefore hypothesize that women prescribed local estrogens were at baseline at higher risk for infections in general, and thus even for COVID-19 (potential indication bias).

It is of note that the majority of COVID-19 fatal events in Sweden among the elderly were observed in care homes and were primarily seen during the first wave of the pandemic, when doctors were not adequately trained to treat COVID-19 effectively [35]. During the same period, the use of protective equipment (facemasks/gloves) for health care workers was not mandatory or even encouraged, increasing their risk of transmitting the disease [36]. Thus, in general, women actually being prescribed local estrogens present with factors rendering them at higher risk for both infection and morbidity due to COVID-19, which could explain our findings.

### Comparison with related studies

Our study findings are in line with that of Costeira et al.; in a population-based cohort from the UK including menopausal women on MHT or other hormonal therapies (e.g., combined oral contraceptive pills, COCPs), the authors demonstrated increased risks of predicted COVID-19 for MHT users alone [13]. The authors did not report on mortality, but no increased risk of hospitalization was seen among MHT users alone which was also confirmed in our study. Unfortunately, Costeira et al. [13] did not collect information on MHT type or route of administration. On the contrary, a handful of studies have suggested a protective effect of MHT use against COVID-19 severity and even the risk of contracting the disease. A recently published study from Sweden included three groups of postmenopausal women, namely women with breast cancer receiving tamoxifen or aromatase inhibitors, women receiving MHT, and control women not receiving estrogen-modulating treatment [12]. In this study, Sund et al. demonstrated a lower adjusted risk of death due to COVID-19 in women under MHT compared to the control group with a reversed relationship between estrogen levels and risk of COVID-19-related death [12]. However, it should be noted that the groups studied by Sund et al. did not arise from the general background population of perimenopausal women but originated instead from the population with laboratory confirmed SARS-CoV-2 infection. In fact, during the first wave of the pandemic in Sweden, immigrants from low- and middle-income countries and/or increased household size contracted the virus to a greater extent and were affected disproportionately by COVID-19 [37]. In line with that, the control group in the study by Sund et al. [12] included a higher proportion of individuals with worse socioeconomic status (i.e., poorest income quintile) and low education level (i.e., primary education), both risk factors that contribute to the increased mortality due to COVID-19 [38–40]. Thus, despite the fact that the authors adjusted for socioeconomical background factors, we believe that the intrinsic risk of the “control population” was already amplified, tipping the risk balance in favor of the study group. On the contrary, the comparison group in our study was matched according to age and healthcare region, and the regression models were also adjusted for education and socioeconomic status, limiting the risk of confounding. Likewise, to Sund et al., a retrospective cohort study from the UK by Dambha-Miller et al. showed that hormone replacement treatment was associated with lower all-cause mortality in COVID-19 positive women (aOR: 0.22, 95% CIs: 0.05–0.94), whereas no associations were found for women receiving COCPs. The latter finding indicated that the difference in COVID-19 mortality might be the result of a greater increase in hormone levels observed in perimenopausal women on MHT compared to premenopausal women on COCPs [14]. Again, the control group chosen was older, socioeconomically deprived, and with a higher rate of comorbidities, while the outcome regarded all-cause mortality and not just COVID-19-specific mortality. In addition, an analysis of electronic health records for a large (*n* = 68,466), international COVID-19 cohort [4] by Seeland et al., demonstrated that women older than 50 years under estradiol treatment had lower fatality risk, by more than 50%, compared to non-treated women (HR: 0.29, 95% CIs: 0.11–0.76) [4]. It should however be noted that the study population originated once more from the infected population probably affecting the intrinsic COVID-19 fatality risk of the control group [4]. Lastly, a multinational, cross-sectional retrospective study from Latin America including mid-aged women attending a routine health check-up reported that women on combined MHT (e.g., containing both estrogen and progestogen) presented a lower risk of laboratory confirmed SARS-CoV-2 infection (OR 0.62, 95% CI 0.41–0.94) [15], which comes in contrast to our findings. However, no association was found between estrogen-only containing MHT and COVID-19 infection, similarly to our study [15]. It should however be noted that participants in the study were relatively affluent (attending private clinical centers), younger (aged 40–64 years), and therefore not representative of the general population. Furthermore, due to the study design, women who experienced more severe COVID-19 disease or persistent symptoms were not included in the population and mortality could not be assessed.

## Strengths and limitations

The main study strength is the fact that we used data from a large nationwide cohort in a country with high COVID-19 incidence during the first and second COVID-19 wave, lowering the risk of selection bias and making results generalizable to the whole population of peri-post menopausal women in Sweden. Moreover, data was obtained from multiple well-validated registries, thus limiting the risk for report bias. Lastly, we had access to a wide range of covariates with a potential role in COVID-19 morbidity and mortality and were therefore able to adjust for several confounders.

The study limitations include lacking information regarding compliance to pharmaceutical treatment such as MHT as well as purchases over the counter during the pandemic (e.g., local estrogens); as a consequence, misclassification cannot be entirely ruled out. Lockdowns were not implemented in Sweden and potential drug shortages during that period of the pandemic would not affect our estimates since these are based on actually dispensed medications before the pandemic outbreak.

Furthermore, we lack data on the menopausal state of the participants since menopause is not always recorded in the register through an ICD-10 diagnosis code. Thus, a proportion of the non-exposed population are expected to be premenopausal and not have the same risk for COVID-19 outcomes as those in peri- and post-menopause exposed to MHT [41]; this proportion, however, of the comparison group is expected to be lower with advancing age. The sources of exposure misclassification above should in any case not have induced false associations but would rather attenuate existing ones. In addition, despite adjusting for a variety of comorbid conditions, residual unmeasured confounding is still possible. Indication bias, especially in the case of those needing treatment with local estrogens vaginally who might have a higher infection susceptibility, cannot be ruled out. We also lacked data on the use of immunosuppressant drugs (such as corticosteroids) that could potentially alter the COVID-19 infection severity. Furthermore, free testing at healthcare facilities was established in the middle of June 2020. Due to somewhat lower testing capacity at the very beginning of the pandemic, there are certainly individuals positive for COVID-19 who were never tested and recorded in the system. This might have introduced some degree of misclassification of this outcome, which nevertheless is expected to be non-differential; this could have led to possible attenuation of some associations. In an effort to account for this, relevant imputations regarding secondary outcome definitions were performed. In addition, we lack information on the BMI of the participating women; we have instead adjusted the regression analyses for the ICD-10 diagnosis of obesity, which has a similar detrimental effect on COVID-19 morbidity and mortality. It should however be noted that registry data only capture the most severe cases, increasing the risk of missing individuals with obesity and incorrectly classifying them as not having the condition. Nevertheless, based on contraindications for MHT use, the misclassification would mostly affect the unexposed population and could have eventually attenuated our findings; it is not expected to have introduced false associations. In addition, initiation of MHT after the study start did not constitute an exclusion criterium per se; however, because of the exposure definition used in the study, women with later dated prescriptions could nevertheless not be included in our analyses, as they were censored after prescription date. Moreover, despite the population-based character of the study, the relative rarity of certain exposures (such as use of bioidentical progestogens) and outcomes restricts our capacity to detect some exposure-outcome associations and thus lowers the study’s statistical power in relation to certain research questions. Lastly, asymptomatic individuals or those with milder symptoms that never sought health care were not captured in this study, affecting the (less severe) secondary outcome events of COVID-19; however, that should not affect the events of the primary outcome (death) nor the severe secondary outcomes (inpatient hospitalization with or without ICU admission).

## Conclusions

In this population-based register study in Sweden, we could not confirm that MHT succeeded in reversing the theoretically increased risks for COVID-19 complications among menopausal women, with indications for use. Initiating or continuing MHT treatment solely for this purpose is therefore advised against. On the contrary, an increased risk for COVID-19 mortality was observed among women on local estrogens compared to non-exposed women. This finding can be due to indication bias and unmeasured confounding, such as frailty and susceptibility for infections among women on local estrogen therapy. Increased risks among older women on systemic estrogens at the start of the study period were no longer apparent when addressing current use. The specific role of MHT cessation for COVID-19-related mortality in this group warrants further investigation. No increased risks for COVID-19 mortality were observed among women on MHT prescribed in ages according to existing clinical recommendations. The findings need to be interpreted with caution, as they were mostly evident during the first pandemic wave and are subject to inherent limitations in register data; nevertheless, they do need to be explored further, in order to guide clinical recommendations.

### Supplementary Information

**Additional file 1: Appendix 1-2. Appendix 1.** Categorization of exposure groups according to their Anatomic Therapeutic Codes (ATC-codes). **Appendix 2.** Comorbidities included in the modified Charlson Comorbidity Index (CCI).

**Additional file 2: Tables S1-S3. Table S1.** Crude and adjusted Cox regression models regarding the association of systemic estrogen treatment with/without progestogens in relation to death due to COVID-19, laboratory-confirmed SARS-CoV-2 infection or outpatient visits/inpatient hospitalizations with/without ICU admission. **Table S2.** Crude and adjusted Cox regression models regarding the association of sex steroid treatment in relation to all-cause mortality. **Table S3.** Crude and adjusted Cox regression models stratified by timing in the COVID-19 pandemic outbreak in relation to the first (1^st^ January 2020–31^st^ August 2020) or second wave of COVID-19 pandemic (1st September 2020–31^st^ December 2020).

## Data Availability

Restrictions apply to the availability of some or all data generated or analyzed during this study to preserve patient confidentiality or because they were used under license. The corresponding author will on request detail the restrictions and any conditions under which access to some data may be provided.

## Abbreviations

CCI: Charlson Comorbidity Index
CI: Confidence interval
COVID-19: Coronavirus disease-19
DDD: Defined daily dose
HR: Hazzard ratio
ICD: International Classification of Diseases
IUD: Intrauterine device
MHT: Menopause hormonal therapy
SARS-CoV-2: Severe acute respiratory syndrome coronavirus 2
