# Supplementary material for: Estrogen-modulating treatment among mid-life women and COVID-19 morbidity and mortality: a multiregister nationwide matched cohort study in Sweden
Source: BMC Med. 2024 Feb 27;22:84. doi: 10.1186/s12916-024-03297-z (PMC10898018; doi:10.1186/s12916-024-03297-z)
Supplement: Supplementary file 1 — Additional file 1: Appendix 1-2. Appendix 1. Categorization of exposure groups according to their Anatomic Therapeutic Codes (ATC-codes). Appendix 2. Comorbidities included in the modified Charlson Comorbidity Index (CCI). [file 12916_2024_3297_MOESM1_ESM.docx]

**Additional file 1: Appendix 1-2**

**Appendix 1: Categorization of exposure groups according to their Anatomic Therapeutic Codes (ATC-codes)**

1. Women on local estrogens alone (estradiol/estriol) (ATC codes G03CA03 or G03CA04).
2. Women on systemic estrogens alone (estradiol/estriol/conjugated estrogens), transdermally or orally, without progestogens (ATC codes G03CA03, G03CA04 or G03CA57).
3. Women on both estrogen AND progestogen either taken separately or combined, in a continuous or sequential manner. The estrogen preparations taken separately included transdermal or oral estradiol (ATC code G03CA03). The progestogens taken separately included hormonal IUD (levonorgestrel) (ATC code G02BA03), vaginal progesterone (progesterone/dydrogesterone) (ATC codes G03DA04 or G03DB01), transdermal synthetic progestin (norethisterone) (ATC codes G03FA01 or G03FB05) or oral synthetic progestin (noretisterone/ lynestrenol/ medroxiprogesterone/ etonogestrel/ desogestrel/ drosperinone) (ATC codes G03AC, G03DA02, G03DB08, G03DC02 or G03DC03). The combined continuous or sequential preparations included oral medications or transdermal patches (ethinylestradiol/ 17 β-estradiol/ conjugated estrogens/ estradiol valerate AND levonorgestrel/ noretisterone/ medroxiprogesterone/ dydrogesterone/ dienogest/ drosperinone/ medrogestone/ nomegestrol/ norgestimate/ norelgestromin) (ATC codes G03AA, G03AB, G03CC07, G03FA01, G03FA12, G03FA14, G03FA15, G03FA17, G03FB05, G03FB06, G03FB07 or G03FB08).
4. Women on progestogens alone without transdermal or oral estrogens; either hormonal IUD (levonorgestrel) (ATC code G02BA03), bioidentical vaginal progesterone alone (progesterone/ dydrogesterone) (ATC codes G03DA04 or G03DB01), oral synthetic progestins alone (noretisterone/ lynestrenol/ medroxiprogesterone/ etonogestrel/ desogestrel/ drosperinone) (ATC codes G03AC, G03DB08, G03DA02, G03DC02 or G03DC03) or injectable progesterone alone (ATC code G03DA04).
5. Women on tibolone alone (ATC code G03CX01).

**Appendix 2: Comorbidities included in the modified Charlson Comorbidity Index(CCI).**

| **Comorbidities** | **ICD-10 *** |
| --- | --- |
| Myocardial infarction | I21.x, I22.x |
| Congestive heart failure | I43.x, I50.x |
| Chronic pulmonary  disease | I27.8, I27.9, J40.x-J47.x, |
| Renal disease | N18.x, N19.x,  Z99.2 |
| Cancer | C50.x-C58.x,  C60.x-C63.x |
