# Supplementary material for: Estrogen-modulating treatment among mid-life women and COVID-19 morbidity and mortality: a multiregister nationwide matched cohort study in Sweden
Source: BMC Med. 2024 Feb 27;22:84. doi: 10.1186/s12916-024-03297-z (PMC10898018; doi:10.1186/s12916-024-03297-z)
Supplement: Supplementary file 2 — Additional file 2: Tables S1-S3. Table S1. Crude and adjusted Cox regression models regarding the association of systemic estrogen treatment with/without progestogens in relation to death due to COVID-19, laboratory-confirmed SARS-CoV-2 infection or outpatient visits/inpatient hospitalizations with/without ICU admission. Table S2. Crude and adjusted Cox regression models regarding the association of sex steroid treatment in relation to all-cause mortality. Table S3. Crude and adjusted Cox regression models stratified by timing in the COVID-19 pandemic outbreak in relation to the first (1st January 2020–31st August 2020) or second wave of COVID-19 pandemic (1st September 2020–31st December 2020). [file 12916_2024_3297_MOESM2_ESM.docx]

**Legends**

**Additional file 2: Tables S1-S3**

**Table S1.** Crude and adjusted Cox regression models regarding the association of systemic estrogen treatment with/without progestogens in relation to death due to COVID-19, laboratory-confirmed SARS-CoV-2 infection or outpatient visits/inpatient hospitalizations with/without ICU admission.

**Table S2.** Crude and adjusted Cox regression models regarding the association of sex steroid treatment in relation to all-cause mortality.

**Table S3.** Crude and adjusted Cox regression models stratified by timing in the COVID-19 pandemic outbreak in relation to the first (1^st^ January 2020 - 31^st^ August 2020) or second wave of COVID-19 pandemic (1^st^ September 2020 - 31^st^ December 2020).

**Table S1.** Crude and adjusted Cox regression models regarding the association of systemic estrogen treatment with/without progestogens in relation to death due to COVID-19, laboratory-confirmed SARS-CoV-2 infection or outpatient visits/inpatient hospitalizations with/without ICU admission.

|  |  | | | | | | |  |
| --- | --- | --- | --- | --- | --- | --- | --- | --- |
|  | **Exposed** | | | **Unexposed** | |  |  | |
| **Exposure** | | **n total** | **n events (%)** | **n total** | **n events (%)** | **Crude HR (95% CI)** | **Adjusted HR (95% CI)^*^** | |
| Systemic estrogens with/without progestogens | | 11541 | 5 | 57701 | 8 | 3.13 (1.02-9.55) | 4.22 (1.37-13.05) | |

Abbreviations: HR, hazard ratio; CI: confidence intervals

^*^Models adjusted for age, civil status, income, education, obesity, alcohol dependence syndrome, and Charlson Comorbidity Index(CCI).

|  | **Laboratory confirmed SARS-CoV-2 infection** | | | | | | | | | **Outpatient visits /Inpatient hospitalizations with/without ICU admission** | | | | | | | | | |
| --- | --- | --- | --- | --- | --- | --- | --- | --- | --- | --- | --- | --- | --- | --- | --- | --- | --- | --- | --- |
|  | **Exposed** | | **Unexposed** | | |  | |  | | **Exposed** | | | **Unexposed** | | |  | |  | |
| **Exposure** | **n total** | **n events (%)** | **n total** | **n events (%)** | **Crude HR (95% CI)** | | **Adjusted HR (95% CI)** | | **n total** | | **n events (%)** | **n total** | | **n events (%)** | **Crude HR (95% CI)** | | **Adjusted HR (95% CI)** | |  |
| Systemic estrogens with/without progestogens | 11541 | 1620 | 57701 | 7826 | 1.04 (0.98-1.01) | | 1.04 (0.98-1.10) | | 11541 | | 195 | 57701 | | 1126 | 0.86 (0.74-1.00) | | 0.90 (0.75-1.05) | |  |
|  |  |  |  |  |  | |  | |  | |  |  | |  |  | |  | |  |

Abbreviations: HR, hazard ratio; CI, confidence intervals; ICU, intensive care unit

^*^Models adjusted for age, civil status, income, education, obesity, alcohol dependence syndrome, and Charlson Comorbidity Index(CCI).

**Table S2.** Crude and adjusted Cox regression models regarding the association of sex steroid treatment with death.

|  |  | | | | | |  |
| --- | --- | --- | --- | --- | --- | --- | --- |
|  | **Exposed** | | **Unexposed** | |  |  | |
| **Exposure** | **n total** | **n events (%)** | **n total** | **n events (%)** | **Crude HR (95% CI)** | **Adjusted HR (95% CI)^*^** | |
| Local estrogens alone | 9981 | 465(4.7) | 49889 | 2316 (4.6) | 1.00(0.91 - 1.11) | 1.00(0.90 - 1.10) | |
| Systemic estrogens without progestogens | 3189 | 20 (0.6) | 15946 | 100 (0.6) | 1.00(0.62 - 1.62) | 0.99(0.61 - 1.60) | |
| Systemic estrogens and progestogens | 8352 | 6 (0.1) | 41755 | 30 (0.1) | 1.00(0.42 - 2.40) | 1.09(0.45 - 2.63) | |
| Progestogens alone | 9323 | 46 (0.5) | 46616 | 230 (0.5) | 1.00(0.73 - 1.37) | 1.30(0.95 - 1.79) | |
| Tibolone alone | 923 | 0 (0.0) | 4615 | 0 (0.0) | NE | NE | |

Abbreviations: HR, hazard ratio; CI: confidence intervals; NE: not estimated.

^*^Models adjusted for age, civil status, income, education, obesity, alcohol dependence syndrome, and Charlson Comorbidity Index(CCI).

**Table S3.** Crude and adjusted Cox regression models stratified by timing in the COVID-19 pandemic outbreak in relation to the first (1^st^ January 2020 - 31^st^ August 2020) or second wave of COVID-19 pandemic (1^st^ September 2020 - 31^st^ December 2020).

| **Death due to COVID-19** |  |  |  |  |
| --- | --- | --- | --- | --- |
|  | **Exposed** | **Unexposed** |  |  |
| **Local estrogens alone** | **n events** | **n events** | **Crude HR (95% CI)** | **Adjusted HR (95% CI)** |
| Wave 1 | 30 | 63 | 2.38(1.54 - 3.68) | 2.12(1.37 – 3.28) |
| Wave 2 | 20 | 51 | 1.96(1.17 - 3.29) | 1.88 (1.12 – 3.17) |
| **Systemic estrogens without progestogens** |  |  |  |  |
| Wave 1 | 4 | 2 | 10.00(1.83 – 54.60) | 9.51 (1.58 – 57.18) |
| Wave 2 | 1 | 2 | 2.50 (0.23-27.60) | 2.07 (0.18-23.39) |
| **Estrogens and progestogens** |  |  |  |  |
| Wave 1 | 0 | 2 | NE | NE |
| Wave 2 | 0 | 2 | NE | NE |
| **Progestogens alone** |  |  |  |  |
| Wave 1 | 2 | 4 | 2.50 (0.46 – 13.65) | 5.46 (0.91 – 32.83) |
| Wave 2 | 0 | 3 | NE | NE |
| **Tibolone alone** |  |  |  |  |
| Wave 1 | 0 | 0 | NE | NE |
| Wave 2 | 0 | 0 | NE | NE |

| **Laboratory confirmed SARS-CoV-2** |  |  |  |  |
| --- | --- | --- | --- | --- |
|  | **Exposed** | **Unexposed** |  |  |
| **Local estrogens alone** | **n events** | **n events** | **Crude HR (95% CI)** | **Adjusted HR (95% CI)** |
| Wave 1 | 313 | 1274 | 1.15 (1.02 - 1.30) | 1.10 (0.97 - 1.25) |
| Wave 2 | 734 | 3285 | 1.15 (1.06 - 1.25) | 1.14 (1.05 - 1.24) |
| **Systemic estrogens without progestogens** |  |  |  |  |
| Wave 1 | 78 | 388 | 0.99 (0.78 - 1.27) | 1.02 (0.80 - 1.30) |
| Wave 2 | 346 | 1824 | 0.95 (0.85 - 1.07) | 0.96 (0.86 - 1.08) |
| **Estrogens and progestogens** |  |  |  |  |
| Wave 1 | 165 | 949 | 0.87 (0.74 - 1.02) | 0.90 (0.76 - 1.07) |
| Wave 2 | 1031 | 4668 | 1.10 (1.03 - 1.18) | 1.10 (1.03 - 1.18) |
| **Progestogens alone** |  |  |  |  |
| Wave 1 | 224 | 1067 | 1.04 (0.90 - 1.20) | 1.04 (0.90 - 1.20) |
| Wave 2 | 1150 | 5542 | 1.04 (0.98 - 1.11) | 1.03 (0.97 - 1.10) |
| **Tibolone alone** |  |  |  |  |
| Wave 1 | 26 | 98 | 1.33 (0.86 - 2.05) | 1.30 (0.84 - 2.01) |
| Wave 2 | 118 | 491 | 1.21 (0.99 - 1.48) | 1.21 (0.99 - 1.48) |

| **Outpatient visits /Inpatient hospitalizations with/without ICU admission** |  |  |  |  |
| --- | --- | --- | --- | --- |
|  | **Exposed** | **Unexposed** |  |  |
| **Local estrogens alone** | **n events** | **n events** | **Crude HR (95% CI)** | **Adjusted HR (95% CI)** |
| Wave 1 | 221 | 915 | 1.26 (1.09 - 1.46) | 1.21 (1.04 - 1.40) |
| Wave 2 | 164 | 612 | 1.42 (1.20 - 1.69) | 1.40 (1.17 - 1.66) |
| **Systemic estrogens without progestogens** |  |  |  |  |
| Wave 1 | 51 | 215 | 1.20 (0.89 - 1.63) | 1.23 (0.90 - 1.67) |
| Wave 2 | 21 | 136 | 0.80 (0.50 - 1.26) | 0.82 (0.52 - 1.30) |
| **Estrogens and progestogens** |  |  |  |  |
| Wave 1 | 74 | 494 | 0.75 (0.59 - 0.96) | 0.82 (0.64 - 1.04) |
| Wave 2 | 49 | 283 | 0.86 (0.64 - 1.17) | 0.96 (0.71 - 1.31) |
| **Progestogens alone** |  |  |  |  |
| Wave 1 | 108 | 570 | 0.95 (0.77 - 1.17) | 0.96 (0.78 - 1.18) |
| Wave 2 | 61 | 351 | 0.90 (0.68 - 1.18) | 0.92 (0.70 - 1.21) |
| **Tibolone alone** |  |  |  |  |
| Wave 1 | 14 | 57 | 1.23 (0.69 - 2.21) | 1.28 (0.71 - 2.30) |
| Wave 2 | 9 | 37 | 1.22 (0.59 - 2.52) | 1.33 (0.64 - 2.77) |

^*^Models adjusted for age, civil status, income, education, obesity, alcohol dependence syndrome, and Charlson Comorbidity Index(CCI).

NE: not estimated.
